# Supplementary material for: COZOID: contact zone identifier for visual analysis of protein-protein interactions
Source: BMC Bioinformatics. 2018 Apr 6;19:125. doi: 10.1186/s12859-018-2113-6 (PMC5889581; doi:10.1186/s12859-018-2113-6)
Supplement: Supplementary file 2 — Software build. Executable binary file of the software tool. (ZIP 118,784 kb) [file 12859_2018_2113_MOESM2_ESM.zip › cozoid/java/docs/beansbinding-1.2.1-doc.zip!/index-files/index-1.html]

A-Index (Beans Binding)


---


|  |  |  |
| --- | --- | --- |
| **Overview** \| Package \| Class \| Use \| **Tree** \| **Deprecated** \| **Index** \| **Help** | |  |
| PREV LETTER   **NEXT LETTER** | **FRAMES**    **NO FRAMES**     **All Classes** |  |


A B C D E F G H I J K L M N O P Q R S T U V W 

---


## **A**

**AbstractBindingListener** - Class in org.jdesktop.beansbinding: An abstract subclass of `BindingListener` that simplifies writing `BindingListeners` by allowing you to extend this class and re-implement only the methods you care about. **AbstractBindingListener()** - Constructor for class org.jdesktop.beansbinding.AbstractBindingListener: **AbstractButtonAdapterProvider** - Class in org.jdesktop.swingbinding.adapters: **AbstractButtonAdapterProvider()** - Constructor for class org.jdesktop.swingbinding.adapters.AbstractButtonAdapterProvider: **AbstractButtonAdapterProvider.Adapter** - Class in org.jdesktop.swingbinding.adapters: **AbstractColumnBinding** - Class in org.jdesktop.swingbinding.impl: **AbstractColumnBinding(int, Property, Property, String)** - Constructor for class org.jdesktop.swingbinding.impl.AbstractColumnBinding: **accept(NodeVisitor)** - Method in interface org.jdesktop.el.impl.parser.Node: **accept(NodeVisitor)** - Method in class org.jdesktop.el.impl.parser.SimpleNode: **add(ELResolver)** - Method in class org.jdesktop.el.CompositeELResolver: Adds the given resolver to the list of component resolvers. **add(Object, Object)** - Static method in class org.jdesktop.el.impl.lang.ELArithmetic: **add(Number, Number)** - Method in class org.jdesktop.el.impl.lang.ELArithmetic: **add(Number, Number)** - Method in class org.jdesktop.el.impl.lang.ELArithmetic.BigDecimalDelegate: **add(Number, Number)** - Method in class org.jdesktop.el.impl.lang.ELArithmetic.BigIntegerDelegate: **add(Number, Number)** - Method in class org.jdesktop.el.impl.lang.ELArithmetic.DoubleDelegate: **add(Number, Number)** - Method in class org.jdesktop.el.impl.lang.ELArithmetic.LongDelegate: **add\_escapes(String)** - Method in exception org.jdesktop.el.impl.parser.ParseException: Used to convert raw characters to their escaped version when these raw version cannot be used as part of an ASCII string literal. **addBinding(Binding)** - Method in class org.jdesktop.beansbinding.BindingGroup: Adds a `Binding` to this group. **addBindingListener(BindingListener)** - Method in class org.jdesktop.beansbinding.Binding: Adds a `BindingListener` to be notified of changes to this `Binding`. **addBindingListener(BindingListener)** - Method in class org.jdesktop.beansbinding.BindingGroup: Adds a `BindingListener` to be notified of all `BindingListener` notifications fired by any `Binding` in the group. **addColumnBinding(Property<E, ?>)** - Method in class org.jdesktop.swingbinding.JTableBinding: Creates a `ColumnBinding` and adds it to the end of the list of `ColumnBindings` maintained by this `JTableBinding`. **addColumnBinding(Property<E, ?>, String)** - Method in class org.jdesktop.swingbinding.JTableBinding: Creates a named `ColumnBinding` and adds it to the end of the list of `ColumnBindings` maintained by this `JTableBinding`. **addColumnBinding(int, Property<E, ?>)** - Method in class org.jdesktop.swingbinding.JTableBinding: Creates a `ColumnBinding` and inserts it at the given index into the list of `ColumnBindings` maintained by this `JTableBinding`. **addColumnBinding(int, Property<E, ?>, String)** - Method in class org.jdesktop.swingbinding.JTableBinding: Creates a `ColumnBinding` and inserts it at the given index into the list of `ColumnBindings` maintained by this `JTableBinding`. **added(int, int)** - Method in class org.jdesktop.swingbinding.impl.ListBindingManager: **addEscapes(String)** - Static method in error org.jdesktop.el.impl.parser.TokenMgrError: Replaces unprintable characters by their espaced (or unicode escaped) equivalents in the given string **addFunction(String, String, Method)** - Method in class org.jdesktop.el.impl.lang.FunctionMapperImpl: **addObservableListListener(ObservableListListener)** - Method in interface org.jdesktop.observablecollections.ObservableList: Adds a listener that is notified when the list changes. **addObservableMapListener(ObservableMapListener)** - Method in interface org.jdesktop.observablecollections.ObservableMap: Adds a listener to this observable map. **addPropertyChangeListener(PropertyChangeListener)** - Method in class org.jdesktop.beansbinding.Binding: Adds a `PropertyChangeListener` to be notified when any property of this `Binding` changes. **addPropertyChangeListener(String, PropertyChangeListener)** - Method in class org.jdesktop.beansbinding.Binding: Adds a `PropertyChangeListener` to be notified when the property identified by the `propertyName` argument changes on this `Binding`. **addPropertyChangeListener(PropertyChangeListener)** - Method in class org.jdesktop.swingbinding.adapters.BeanAdapterBase: **addPropertyChangeListener(String, PropertyChangeListener)** - Method in class org.jdesktop.swingbinding.adapters.BeanAdapterBase: **addPropertyStateListener(S, PropertyStateListener)** - Method in class org.jdesktop.beansbinding.ObjectProperty: Does nothing; the state of an `ObjectProperty` never changes so listeners aren't useful. **addPropertyStateListener(S, PropertyStateListener)** - Method in class org.jdesktop.beansbinding.Property: Adds a `PropertyStateListener` to be notified when the state of the `Property` changes with respect to the given source. **addPropertyStateListener(S, PropertyStateListener)** - Method in class org.jdesktop.beansbinding.PropertyHelper: Adds a `PropertyStateListener` to be notified when the state of the `Property` changes with respect to the given source. **adjustBeginLineColumn(int, int)** - Method in class org.jdesktop.el.impl.parser.SimpleCharStream: Method to adjust line and column numbers for the start of a token. **allChanged()** - Method in class org.jdesktop.swingbinding.impl.ListBindingManager: **And()** - Method in class org.jdesktop.el.impl.parser.ELParser: **AND0** - Static variable in interface org.jdesktop.el.impl.parser.ELParserConstants: **AND1** - Static variable in interface org.jdesktop.el.impl.parser.ELParserConstants: **ArithmeticNode** - Class in org.jdesktop.el.impl.parser: **ArithmeticNode(int)** - Constructor for class org.jdesktop.el.impl.parser.ArithmeticNode: **ArrayELResolver** - Class in org.jdesktop.el: Defines property resolution behavior on arrays. **ArrayELResolver()** - Constructor for class org.jdesktop.el.ArrayELResolver: Creates a new read/write `ArrayELResolver`. **ArrayELResolver(boolean)** - Constructor for class org.jdesktop.el.ArrayELResolver: Creates a new `ArrayELResolver` whose read-only status is determined by the given parameter. **AstAnd** - Class in org.jdesktop.el.impl.parser: **AstAnd(int)** - Constructor for class org.jdesktop.el.impl.parser.AstAnd: **AstBracketSuffix** - Class in org.jdesktop.el.impl.parser: **AstBracketSuffix(int)** - Constructor for class org.jdesktop.el.impl.parser.AstBracketSuffix: **AstChoice** - Class in org.jdesktop.el.impl.parser: **AstChoice(int)** - Constructor for class org.jdesktop.el.impl.parser.AstChoice: **AstCompositeExpression** - Class in org.jdesktop.el.impl.parser: **AstCompositeExpression(int)** - Constructor for class org.jdesktop.el.impl.parser.AstCompositeExpression: **AstDeferredExpression** - Class in org.jdesktop.el.impl.parser: **AstDeferredExpression(int)** - Constructor for class org.jdesktop.el.impl.parser.AstDeferredExpression: **AstDiv** - Class in org.jdesktop.el.impl.parser: **AstDiv(int)** - Constructor for class org.jdesktop.el.impl.parser.AstDiv: **AstDotSuffix** - Class in org.jdesktop.el.impl.parser: **AstDotSuffix(int)** - Constructor for class org.jdesktop.el.impl.parser.AstDotSuffix: **AstDynamicExpression** - Class in org.jdesktop.el.impl.parser: **AstDynamicExpression(int)** - Constructor for class org.jdesktop.el.impl.parser.AstDynamicExpression: **AstEmpty** - Class in org.jdesktop.el.impl.parser: **AstEmpty(int)** - Constructor for class org.jdesktop.el.impl.parser.AstEmpty: **AstEqual** - Class in org.jdesktop.el.impl.parser: **AstEqual(int)** - Constructor for class org.jdesktop.el.impl.parser.AstEqual: **AstFalse** - Class in org.jdesktop.el.impl.parser: **AstFalse(int)** - Constructor for class org.jdesktop.el.impl.parser.AstFalse: **AstFloatingPoint** - Class in org.jdesktop.el.impl.parser: **AstFloatingPoint(int)** - Constructor for class org.jdesktop.el.impl.parser.AstFloatingPoint: **AstFunction** - Class in org.jdesktop.el.impl.parser: **AstFunction(int)** - Constructor for class org.jdesktop.el.impl.parser.AstFunction: **AstGreaterThan** - Class in org.jdesktop.el.impl.parser: **AstGreaterThan(int)** - Constructor for class org.jdesktop.el.impl.parser.AstGreaterThan: **AstGreaterThanEqual** - Class in org.jdesktop.el.impl.parser: **AstGreaterThanEqual(int)** - Constructor for class org.jdesktop.el.impl.parser.AstGreaterThanEqual: **AstIdentifier** - Class in org.jdesktop.el.impl.parser: **AstIdentifier(int)** - Constructor for class org.jdesktop.el.impl.parser.AstIdentifier: **AstInteger** - Class in org.jdesktop.el.impl.parser: **AstInteger(int)** - Constructor for class org.jdesktop.el.impl.parser.AstInteger: **AstLessThan** - Class in org.jdesktop.el.impl.parser: **AstLessThan(int)** - Constructor for class org.jdesktop.el.impl.parser.AstLessThan: **AstLessThanEqual** - Class in org.jdesktop.el.impl.parser: **AstLessThanEqual(int)** - Constructor for class org.jdesktop.el.impl.parser.AstLessThanEqual: **AstLiteralExpression** - Class in org.jdesktop.el.impl.parser: **AstLiteralExpression(int)** - Constructor for class org.jdesktop.el.impl.parser.AstLiteralExpression: **AstMinus** - Class in org.jdesktop.el.impl.parser: **AstMinus(int)** - Constructor for class org.jdesktop.el.impl.parser.AstMinus: **AstMod** - Class in org.jdesktop.el.impl.parser: **AstMod(int)** - Constructor for class org.jdesktop.el.impl.parser.AstMod: **AstMult** - Class in org.jdesktop.el.impl.parser: **AstMult(int)** - Constructor for class org.jdesktop.el.impl.parser.AstMult: **AstNegative** - Class in org.jdesktop.el.impl.parser: **AstNegative(int)** - Constructor for class org.jdesktop.el.impl.parser.AstNegative: **AstNot** - Class in org.jdesktop.el.impl.parser: **AstNot(int)** - Constructor for class org.jdesktop.el.impl.parser.AstNot: **AstNotEqual** - Class in org.jdesktop.el.impl.parser: **AstNotEqual(int)** - Constructor for class org.jdesktop.el.impl.parser.AstNotEqual: **AstNull** - Class in org.jdesktop.el.impl.parser: **AstNull(int)** - Constructor for class org.jdesktop.el.impl.parser.AstNull: **AstOr** - Class in org.jdesktop.el.impl.parser: **AstOr(int)** - Constructor for class org.jdesktop.el.impl.parser.AstOr: **AstPlus** - Class in org.jdesktop.el.impl.parser: **AstPlus(int)** - Constructor for class org.jdesktop.el.impl.parser.AstPlus: **AstString** - Class in org.jdesktop.el.impl.parser: **AstString(int)** - Constructor for class org.jdesktop.el.impl.parser.AstString: **AstTrue** - Class in org.jdesktop.el.impl.parser: **AstTrue(int)** - Constructor for class org.jdesktop.el.impl.parser.AstTrue: **AstValue** - Class in org.jdesktop.el.impl.parser: **AstValue(int)** - Constructor for class org.jdesktop.el.impl.parser.AstValue: **AstValue.Target** - Class in org.jdesktop.el.impl.parser: **AstValue.Target()** - Constructor for class org.jdesktop.el.impl.parser.AstValue.Target: **AutoBinding**<SS,SV,TS,TV> - Class in org.jdesktop.beansbinding: An implementation of `Binding` that automatically syncs the source and target by refreshing and saving according to one of three update strategies. **AutoBinding(AutoBinding.UpdateStrategy, SS, Property<SS, SV>, TS, Property<TS, TV>, String)** - Constructor for class org.jdesktop.beansbinding.AutoBinding: Create an instance of `AutoBinding` between two properties of two objects, with the given update strategy. **AutoBinding.UpdateStrategy** - Enum in org.jdesktop.beansbinding: An enumeration representing the possible update strategies of an `AutoBinding`.

---


|  |  |  |
| --- | --- | --- |
| **Overview** \| Package \| Class \| Use \| **Tree** \| **Deprecated** \| **Index** \| **Help** | |  |
| PREV LETTER   **NEXT LETTER** | **FRAMES**    **NO FRAMES**     **All Classes** |  |


A B C D E F G H I J K L M N O P Q R S T U V W 

---
